# A critical evaluation of the content validity of patient-reported outcome measures assessing health-related quality of life in children with cancer: a systematic review

**DOI:** 10.1186/s41687-023-00540-8

**Published:** 2023-01-19

**Authors:** Maria Rothmund, Andreas Meryk, Gerhard Rumpold, Roman Crazzolara, Samantha Sodergren, Anne-Sophie Darlington, David Riedl, the EORTC Quality of Life Group

**Affiliations:** 1 Department of Psychiatry, Psychotherapy, Psychosomatics and Medical Psychology, University Clinic of Psychiatry II, Medical University Innsbruck, Innsbruck, Austria; 2 Institute of Psychology, University of Innsbruck, Innsbruck, Austria; 3 Department of Pediatrics I, Medical University Innsbruck, Innsbruck, Austria; 4 School of Health Sciences, University of Southampton, Southampton, UK; 5 Ludwig Boltzmann Institute for Rehabilitation Research, Vienna, Austria

**Keywords:** Patient-reported outcomes, Content validity, Questionnaire development, Pediatric oncology, Childhood cancer, Psychometrics, Patient-reported outcome measures

## Abstract

**Background:**

With increasing survival rates in pediatric oncology, the need to monitor health-related quality of life (HRQOL) is becoming even more important. However, available patient-reported outcome measures (PROMs) have been criticized. This review aims to systematically evaluate the content validity of PROMs for HRQOL in children with cancer.

**Methods:**

In December 2021, a systematic literature search was conducted in PubMed. PROMs were included if they were used to assess HRQOL in children with cancer and had a lower age-limit between 8 and 12 years and an upper age-limit below 21 years. The COSMIN methodology for assessing the content validity of PROMs was applied to grade evidence for relevance, comprehensiveness, and comprehensibility based on quality ratings of development studies (i.e., studies related to concept elicitation and cognitive interviews for newly developed questionnaires) and content validity studies (i.e., qualitative studies in new samples to evaluate the content validity of existing questionnaires).

**Results:**

Twelve PROMs were included. Due to insufficient patient involvement and/or poor reporting, the quality of most development studies was rated ‘doubtful’ or ‘inadequate’. Few content validity studies were available, and these were mostly ‘inadequate’. Following the COSMIN methodology, evidence for content validity was ‘low’ or ‘very low’ for almost all PROMs. Only the PROMIS Pediatric Profile had ‘moderate’ evidence. In general, the results indicated that the PROMs covered relevant issues, while results for comprehensiveness and comprehensibility were partly inconsistent or insufficient.

**Discussion:**

Following the COSMIN methodology, there is scarce evidence for the content validity of available PROMs for HRQOL in children with cancer. Most instruments were developed before the publication of milestone guidelines and therefore were not able to fulfill all requirements. Efforts are needed to catch up with methodological progress made during the last decade. Further research should adhere to recent guidelines to develop new instruments and to strengthen the evidence for existing PROMs.

**Supplementary Information:**

The online version contains supplementary material available at 10.1186/s41687-023-00540-8.

## Background

In recent decades, survival rates in pediatric oncology have increased considerably [1–3]. Even though overall survival remains the primary outcome [4], patients’ health-related quality of life (HRQOL) also needs careful monitoring and management. HRQOL as defined by the World Health Organization (WHO) is an “individual’s perception of their position in life […] incorporating in a complex way individuals’ physical health, psychological state, level of independence, social relationships, personal beliefs and their relationships to salient features” [5]. Depending on context and target population, different aspects are relevant for HRQOL. For children with cancer, Anthony et al. [6] have provided the most comprehensive conceptual framework so far. It covers four major domains: physical (symptoms, physical functioning), psychological (emotional distress, behavior, positive psychological function, self-esteem, body image, cognitive health), social (relationships, social functioning), and general health (health perception) [6].

In clinical routine and research, HRQOL is commonly assessed by patient-reported outcome measures (PROMs). In pediatrics, PROMs are often complemented with caregiver-reports. However, patient- and caregiver-reports often differ, especially for less observable outcomes that are only accessible from patient perspective (e.g., perceived burden, satisfaction with relationships) [7–12]. Several studies have indicated that children from 8 years onwards can reliably self-report [13–15]. Thus, it is recommended to treat patient-reports as the most important source of information in this age-group [7, 16]. This is in line with a trend towards increasing the involvement and empowerment of children in research and treatment [17–19].

To assess HRQOL from children’s perspective, evidence-based and age-appropriate PROMs are needed that meet psychometric quality criteria [20]. The most fundamental measurement property is content validity, defined as “the degree to which the content […] is an adequate reflection of the construct(s) to be measures” [20]. Claims regarding content validity can only be made when an instrument *comprehensively* assesses *relevant* aspects in a *comprehensible* way [21, 22].

To ensure content validity, PROM development guidelines strongly recommend patient involvement in several stages [15, 21, 23–26]. They suggest involving patients in concept elicitation and issue generation to give their opinion on relevance and comprehensiveness. Later in the process, guidelines request cognitive interviews to evaluate whether item formulations, response-options, and recall-periods are understood as intended.

For children from the age of 8 years, recall-periods from 7 days to 4 weeks and faces-scales with ≤ 6 faces or Likert-scales with ≤ 5 points are usually considered suitable [24, 27]. Adolescents and young adults (AYAs) around 14 years or older can complete the same tools as adults [28], but they face distinct HRQOL issues as they transition into adulthood [29, 30].

Previous research has indicated that children with cancer were insufficiently involved in the development of existing PROMs [31]. It has been questioned whether they measure what is relevant for children [32], and whether they are complete [33] and of sufficient psychometric quality [31, 34].

The present systematic review aims to systematically evaluate the content validity of available PROMs for HRQOL in children with cancer aged between 8 and 14 years. To do so, the COSMIN methodology for assessing the content validity of PROMs [21, 22; COSMIN = COnsensus-based Standards for the selection of health Measurement INstruments] is applied. In a recently published review, this methodology was used to evaluate PROMs measuring positive psychological constructs [35]. Previous reviews using the COSMIN methodology to evaluate PROMs for pediatric oncology [34, 36, 37] were based on an older version [38–40], which was less comprehensive. The previous COSMIN guideline did not cover the key concept of comprehensibility, and its standards only checked whether certain steps were undertaken, without evaluating the methodological quality [22]. Thus, it is expected that ratings based on the old version will vary considerably from ratings based on the current version.

## Methods

This systematic review follows the Preferred Reporting Items of Systematic Reviews and Meta-analyses (PRISMA) guidelines, where applicable [41]. The PRISMA checklist is provided in Additional file 1. At the time when we started to work on this review, it was not possible to register the protocol since common platforms (e.g., PROSPERO) accepted COVID-19-related protocols only. Thus, no protocol has been published.

### Search strategy and study selection

A literature search was conducted on PubMed in December 2021 combining Medical Subject Headings (MeSH) related to HRQOL, the target population of children with cancer, and psychometrics: *(“Quality of Life”[MeSH] AND (Neoplasms [MeSH] OR “Medical Oncology”[MeSH]) AND (Child [MeSH] OR Pediatrics [MeSH]) AND ("Self Assessment"[MeSH] OR "Patient Reported Outcome Measures"[MeSH] OR "Patient Outcome Assessment"[MeSH] OR "Self Report"[MeSH] OR "Psychometrics"[MeSH]))*. The search was neither limited to a specific time-period nor filtered for specific languages.

As a first step, abstracts were screened by one reviewer [MR] to identify PROMs for HRQOL assessment used in children with cancer within the age range between 8 and 14 years. This included generic and cancer-specific instruments but excluded survivor-specific instruments. PROMs primarily addressing adolescents (lower age-limit at ≥ 12) were excluded, but PROMs for transitional age-groups (children and adolescents) were included if the upper age-limit did not exceed 21 years. A PROM was considered relevant if the developers claimed to assess HRQOL or if it covered physical, psychological, and social health, as described in the conceptual framework by Anthony et al. [6]. PROMs assessing single symptoms or adverse effects were excluded (e.g., PedsQL Fatigue scale [42] or separate PROMIS-scales [43]).

To ensure that all relevant PROMs were included, the list of PROMs was compared to a list of 112 instruments identified by Algurén et al. for the development of the Overall Pediatric Health Standard Set (OPH-SS) [44] and a list of 155 PROMs collected in a simultaneously conducted review of HRQOL issues in children with cancer [45]. For all included instruments, manuals and review copies were searched. If not accessible, authors were contacted. Data regarding their main characteristics were extracted [MR], i.e., the target population (age, diagnoses), recall-period, response-options, the number of items, and the intended scale structure as well as whether a parent-version was available (see Table 1).

**Table 1:** Main characteristics of the included Patient-Reported Outcome Measures (PROMs)

PROM	Target population	Parent-version	Recall period	Response options	Intended scale structure Scale name (number of items per scale)^a^	Studies taken into account
DISABKIDS 12/37	8–16 Chronic Disease	Yes	4 weeks	5-point Likert-scale	Physical limitation (2/6) Treatment (2/6) Independence (2/6) Emotions (2/7) Social exclusion (2/6) Social inclusion (2/6)	[52–55, 68–71]
KIDSCREEN 10/27/52	8–18 Generic	Yes	Last week	5-point Likert-scale	KIDSCREEN 10: Unidimensional trait (10) Overall health perception (1)	[47, 48, 72–74]
					KIDSCREEN 27: Physical well-being (5) Psychological well-being (7) Autonomy and parents (7) Social support and peers (4) School environment (4)	
					KIDSCREEN 52: Physical well-being (5) Psychological well-being (6) Moods and emotions (7) Self-perception (5) Autonomy (5) Parent relations and home (6) Social support and peers (6) School environment (6) Social acceptance/bullying (3) Financial resources (3)	
KINDL-R Generic Kid	7–13 Generic	Yes	Past week	5-point Likert-scale	Physical (4) Emotional (4) Self-esteem (4) Family (4) Friends (4) School (4)	[49, 50, 75–77]
KINDL-R Oncology Module Kid	7–17 Cancer	No	Past week	3-/4-/5-point Likert-scale	Physical well-being (4) Psychological well-being (4) Friends (3) School (2) Treatment (11)	[60]
PAC-QoL Child (provisional)	8–12 Advanced cancer	No	Last week	4-point Likert-scale	Physical comfort (?) Psychological well-being (?) Social interaction (?) Resilience (?) Quality of care (?)	[61, 62]
PedsQL Brain Tumor Module Child	8–12 Brain Tumor	Yes	7 days	5-point Likert-scale	Cognitive problems (7) Pain and hurt (3) Movement and balance (3) Procedural anxiety (3) Nausea (5) Worry (3)	[63, 78–81]
PedsQL Cancer Module 3.0 Child	8–12 Cancer	Yes	one month	5-point Likert-scale	Pain and hurt (2) Nausea (5) Procedural anxiety (3) Treatment anxiety (3) Worry (3) Cognitive problems (5) Perceived physical appearance (3) Communication (3)	[42, 78–80]
PedsQL Generic Core Scale 4.0 Child	8–12 Generic	Yes	one month	5-point Likert-scale	Health and activities (8) Feelings (5) Social functioning (5) School (5)	[42, 51, 78–80, 82–84]
PROMIS v2.0 Pediatric Profile 25/37/49	8–17 Generic and/or chronic Disease	Yes	7 days	5-point Likert-scale	Physical function mobility (4/6/8) Anxiety (4/6/8) Depressive symptoms (4/6/8) Fatigue (4/6/8) Peer relationships (4/6/8) Pain interference (4/6/8) Pain intensity (1)	[56, 57, 85–99]
QOLCC-7-12	7–12 Cancer	Yes	N/A	N/A	Physical function (5) Psychological function (6) Social function (7) Treatment/symptoms (6) Cognitive function (6) Understanding illness (4) Patient-communication (6)	[64, 65, 100, 101]
SQOLPOP	7–12 Cancer	Yes	N/A	N/A	N/A	[66, 67]
TACQOL	6–15 Chronic Disease	Yes	2 weeks	4-point Likert-scale	Body (8) Motor (8) Autonomy (8) Cognition (8) Social (8) Positive emotions (8) Negative emotions (8)	[58, 59, 102]

(?) not unambiguously clear which—and therefore how many—items belong to which (sub)scale

N/A: No information available

^a^Numbers separated by slash: numbers of items per scale for different length versions; sorted from the shortest version to the longest version

In a second step, full-texts and their reference-lists were screened by one reviewer [MR] to identify development and content validity studies for the investigated PROMs. The inclusion and exclusion criteria were based on the definitions provided by the COSMIN guidelines: Development studies include all studies on concept elicitation and studies testing PROMs under development, e.g., cognitive interview studies. Content validity studies include all studies that investigate the relevance, comprehensiveness, and/or comprehensibility of existing PROMs in a new sample. Additional searches on PubMed were conducted with PROM-names and “develop*” or “content valid*” to check whether further relevant studies were available. The included studies were evaluated according to the COSMIN guidelines (see below).

### The COSMIN methodology for assessing content validity

The COSMIN methodology for assessing content validity is divided into three so-called ‘boxes’ with several ‘standards’ [22, 46]. Box 1 evaluates the quality of PROM development, including general design (definition of construct, target population, and context/purpose; 35 standards), concept elicitation (7 standards), and cognitive interviews (22 standards).

Box 2 evaluates the quality of content validity studies, defined as studies on the relevance, comprehensiveness, and comprehensibility of existing PROMs performed in new samples [22]. The standards in box 2 assess whether and how patients were asked about relevance (standards 1–7), comprehensiveness (standards 8–14), and comprehensibility (standards 15–21), and whether and how professionals were asked about relevance (standards 22–26) and comprehensiveness (standards 27–31). As caregivers play an important intermediary role in pediatrics, we wanted to take their input into account as well. After consulting with the COSMIN Group, we decided to use the standards for expert involvement (standards 22–31) to rate whether and how caregivers were asked about relevance and comprehensiveness.

In box 3, the results of development and content validity studies are rated against ten criteria for good content validity. Additionally, reviewers were asked to give their own ratings of comprehensiveness, relevance, and comprehensibility of the tool (eight standards). In terms of comprehensibility, ratings for response-options and recall-periods were based on recommendations from a recent review by Coombes et al. [27]. Item-formulations were rated positive, except if items appeared obviously inappropriate for children. For consistent relevance and comprehensiveness ratings, the items of all PROMs were systematically categorized by content, as described below.

In a final step, the overall ratings are summarized and the quality of evidence is graded. Following the COSMIN guidelines, evidence is rated ‘low’ or ‘very low’ if there has been no content validity study of at least ‘doubtful’ quality. If content validity has not been sufficiently assessed, the development process needs to be of ‘adequate’ or ‘very good’ quality to obtain a ‘moderate’ evidence level. For evidence to obtain a ‘high’ rating, there needs to have been at least one content validity study of ‘adequate’ or ‘very good’ quality.

The ratings of boxes 1 and 2 were conducted by two reviewers independently [MR, AM], using the Excel-sheet available from the COSMIN website (cosmin.nl). We made minor adaptations to this sheet by adding columns for the reviewers to justify their decisions. Conflicts were discussed until consensus was reached. The ratings of box 3 and the final evidence grading were performed by one reviewer [MR] and approved by all co-authors.

### Categorizing items by the contents assessed

To provide a uniform and solid basis for reviewers’ ratings of comprehensiveness and relevance, items from all investigated PROMs were extracted into an Excel-file and mapped onto the conceptual framework by Anthony et al. [6]. Within this hierarchical framework, the domains of physical, psychological, and social health were further divided into subdomains, containing several identifying concepts. For example, physical health is divided into symptoms (e.g., pain, fatigue) and physical function (e.g., dexterity, mobility), while social health is divided into relationships (e.g., with family or peers) and social function (e.g., recreation and leisure, school). The psychological domain has the most subdomains and is divided into emotional distress (e.g., afraid, sad), behavior (e.g., clingy, defiant), positive psychological function (e.g., benefit finding), self-esteem (e.g., feeling loved or proud), body image (e.g., personal appearance), and cognitive issues (e.g., attention, remembering).

Each item was assigned to one domain, subdomain, and identifying concept by one reviewer [MR]. Open-ended questions, conditional items (filter-questions), and determinant questions (on background information of the patient) were not taken into account. To enable a consistent categorization across all items, we defined categorization rules (Additional file 2). A second reviewer [DR] indicated his (dis)agreement per item. Conflicts were discussed until consensus was reached. Where necessary, new subdomains and identifying concepts were added to complement the conceptual framework (Additional file 3).

Descriptive statistics were applied to investigate the representation of contents within the overall item pool and the questionnaires. Item content was considered relevant if it could be assigned to one of the subdomains. Questionnaires were considered comprehensive when they covered physical health and social health (at least family/general) and several aspects of psychological health, i.e., negative emotional health issues (emotional distress or treatment burden), positive issues (positive psychological functioning or self-esteem), and cognitive issues.

## Results

### Identification of PROMs and their main characteristics

**Fig. 1:**
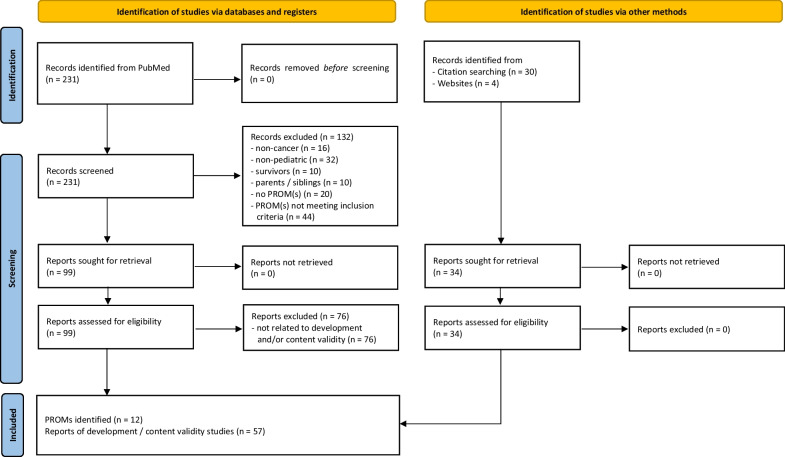
PRISMA 2020 flow diagram of articles selection process. From: Page et al. [41]

As shown in Fig. 1, the literature search identified 231 articles and screening for PROMs resulted in a list of nine inventories (i.e. measurement systems / questionnaire providers). Two of them provided different modules (e.g., generic and cancer-specific), resulting in 12 different PROMs. Taking versions of different length into account, 17 questionnaires were identified. Counterchecking against the PROMs collected for the development of the OPH-SS [44] and our review of HRQOL issues [45] did not yield any additional instruments. For the included PROMs, 53 development and content validity studies and four manuals were identified that were taken into account in the present evaluation (Table 1).

Among the 12 PROMs, three are generic instruments (KIDSCREEN [47, 48], KINDL-R Kid Generic [49, 50], PedsQL Generic Core Scale [42, 51]), another three are for chronically ill children (DISABKIDS [52–55], PROMIS Pediatric Profile [56, 57], and TACQOL-CF [58, 59]), and six are cancer-specific (KINDL-R Kid Oncology Module [60], PAC-QoL Child [61, 62], PedsQL Brain Tumor [63], PedsQL Cancer Module [42], QOLCC [64, 65], SQOLPOP [66, 67]). Among the latter, one is specifically for children with advanced cancer (PAC-QoL), and another is for children with brain tumors (PedsQL Brain Tumor). Further characteristics are presented in Table 1.

### Contents assessed by included PROMs

For all but one PROM (SQOLPOP), review copies or item lists were found. Four-hundred different items were retrieved, some of which belong to more than one length-version or module. Of these 400 items, 22 were excluded as open-ended questions, determinant, or conditional items. No conflicts occurred in defining the question type.

The remaining 378 items were assigned to one of the domains, subdomains, and identifying concepts within the conceptual framework by Anthony et al. [6]. The reviewers agreed upon the categorization of 94.97% of items (359/378). The few conflicts were easily resolved, and the complementation of the HRQOL model for content categorization was discussed [MR, DR] (Additional file 3). The categorizations were adapted accordingly [MR], and the final categorization was approved again [DR].

**Fig. 2:**
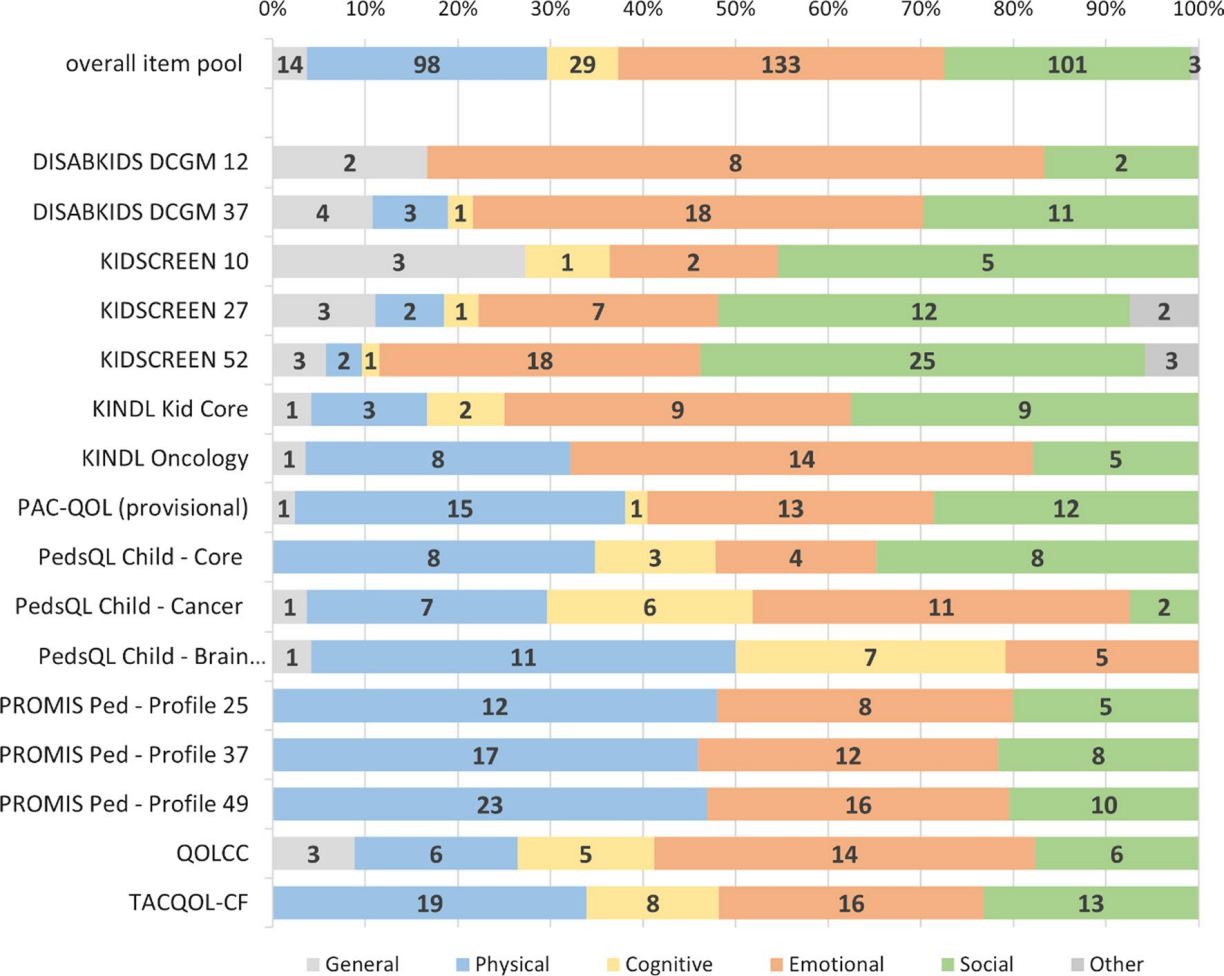
Proportion and total number of items assessing the domains of health-related quality of life within the overall item pool and within the different questionnaires (Numbers in bars indicate the total number of items; length of bars indicates the proportion, compared to the legend above)

Most items from the overall item pool cover psychological aspects. As displayed in Fig. 2, 35.19% (N = 133) of items address emotional health and another 7.67% (N = 19) refer to cognitive health. A quarter of items assess social (N = 191, 26.72%) and physical health (N = 89, 25.93%). Less than 5% measure general health perception or other aspects (i.e., financial).

Upon closer inspection of the different PROMs (Fig. 2), it is apparent that the generic instruments and core scales (except for the PedsQL Generic Core Scale) assess less physical and more social issues than instruments designed for children with chronic diseases or cancer. In contrast, the PROMIS Pediatric Profile and the PedsQL Brain Tumor Module have the strongest focus on physical health, with approximately 50% of their items being dedicated to this domain. Cognitive issues are mostly represented in the PedsQL Brain Tumor and Cancer Modules, but not covered in the PROMIS Pediatric Profile. Additional file 4 provides more detail.

### Quality ratings of development studies

The ratings obtained for the quality of development studies are displayed in Table 2, including justifications for ratings other than ‘very good’ (V). For most instruments, a clear definition of the construct to be measured, the target population, and the context was given. For the KINDL-R Oncology module, these points remained ‘doubtful’, as no development study was available. The SQOLPOP obtained an ‘inadequate’ rating, because the development study did not clarify which dimensions this questionnaire should capture [67].

**Table 2:** Quality ratings of development studies following the COSMIN methodology

PROM	PROM development													Total PROM development
	PROM design and concept elicitation (CE)							Justification for given ratings other than ‘very good’ (V)	Cognitive interviews (CI)				Justification for given ratings other than ‘very good’ (V)	
	General design requirements (GDR)					CE^1^	Total PROM design		GDR	Comprehensibility	Comprehensiveness	Total CI study		
	Construct	Origin of construct	Target population	Context of use	Developed in target population				Performed in target population					
DISABKIDS	V	V	V	V	V	D	**D**	Focus groups and individual interviews were conducted; however, information on experience and skills of group moderators/interviewers is missing [69].	V	D	N/A	**D**	Difficulties of children noticed; however, these were not considered in item reduction/reformulation process, which was only guided by quantitative data and expert consensus [52, 53, 69].	**D**
KIDSCREEN	V	V	V	V	V	V	**V**		V	I	N/A	**I**	Pilot-testing of 185 preliminary items resulted in adaptations; however, items were not tested in their final form and analysis and results were only described vaguely [48, 73].	**I**
KINDL-R Generic	V	V	V	V	V	D	**D**	Item pool developed from literature and discussions with children, but methods not clearly described [75, 76]. Missing information about development of revised version (KINDL-R); studies cited do not contain further information ([76] referred to in [49]) or are not accessible (habilitation thesis referred to in [77]).	V	D	N/A	**D**	Pre-test on comprehensibility of preliminary version conducted, but only healthy children (N = 28) and teachers, only written information, and revisions re-evaluated with only three healthy children [75].	**D**
KINDL-R Oncology	D	D	V	D	I	N/A	**I**	No published development study available. When approached for literature on this module, the responsible office of QOL measures at the university clinic Hamburg-Eppendorf (UKE) referred to the study by Ergin et al. [60] on developing a Turkish translation (mail, 04/16/2020). However, this study does not report anything about the development of the original module.	N/A	N/A	N/A	**I**	No CI study available.	**I**
PAC-QoL Child	V	V	V	V	I	N/A	**I**	No children were included in concept elicitation. For ethical reasons, Cataudella et al. [61] decided to only interview bereaved parents and HCPs at this stage.	V	D	N/A	**D**	CIs indicated that younger children had considerable difficulties in understanding, which has not been adequately addressed so far [62].	**I**
PedsQL Generic	V	V	V	V	A	D	**D**	No clear information is available on how the PedsQL was derived from the previous PCQL [see also 31]. Brief descriptions of PCQL development do not provide sufficient information on sample and methods [79, 80, 82].	N/A	N/A	N/A	**I**	No CIs conducted for the latest version(s) of the PedsQL Generic Core Scale.	**I**
PedsQL Cancer	V	V	V	V	A	D	**D**	Again, missing information on how the PedsQL was derived from PCQL [see also 31].	N/A	N/A	N/A	**I**	NO CI study available.	**I**
PedsQL Brain Tumor	V	V	V	V	A	D	**D**	Palmer et al. [63] state that it was “developed through focus groups with healthcare providers, children and parents, cognitive interviews, pre-testing, and field testing protocols.” (p. 288). Thus, it is assumable that children of the target population were involved. However, as no further information is given, the quality remains ‘doubtful’.	D	D	D	**D**	Missing information results in ‘doubtful’ ratings, see statement by Palmer et al. [63] provided as justification for rating of development studies.	**D**
PROMIS Ped Profile	*per scale*	V	V	V	V	V	**V**		V	A	A	**A**	Even though very good methods applied, rating off CIs can only be ‘adequate’, because 93% of the items have been tested in 5 or 6 patients only [85], which is insufficient to obtain a ‘very good’ rating in the COSMIN manual (7 required). Additional items developed by Quinn et al. [89] have been tested in at least 5 children per item. Further item reduction relied on quantitative methods only (methods described in [103]; results per domain: [87, 90–93].	**A**
*Anxiety and depression*^2^	V	“	“	“	“	“	“		“	“	“	“		“
*Fatigue*	V	“	“	“	“	“	“		“	“	“	“		“
*Mobility and upper extremity*^2^	V	“	“	“	“	“	“		“	“	“	“		“
*Pain intensity*	V	“	“	“	“	“	“		“	“	“	“		“
*Pain interference*	V	“	“	“	“	“	“		“	“	“	“		“
*Peer relationships*	V	“	“	“	“	“	“		“	“	“	“		“
QOLCC-7-12	V	V	V	V	V	I	**I**	The qualitative methods applied were mostly ‘adequate’ to ‘very good’ [101]. ‘Inadequate’ rating because data were not rated by two independent investigators (Yeh was the sole author). However, it must be acknowledged that the results were discussed several times with faculty members, nursing students and participants, i.e., pediatric cancer patients.	D	D	D	**D**	Only three children were included in CIs and no further information is available on methods or results [64].	**I**
SQOLPOP	I	V	V	V	I	N/A	**I**	Kudubes and Bektas [67] state that after a “literature review, dimensions were formed to determine the quality of life from all aspects and item pools were developed for these dimensions” (p. 524), without naming these dimensions at any point. Thus, the definition of the construct to be measured was rated ‘inadequate’. No children were involved in the concept elicitation.	D	D	D	**D**	Pilot-study conducted with 25 children and their parents, who apparently had no negative feedback to give [67]. However, missing information on sample and methods results in ‘doubtful’ rating.	**I**
TACQOL	V	V	V	V	I	N/A	**I**	No children involved in concept elicitation and issue generation.	N/A	N/A	N/A	**I**	Pilot-testing of provisional item list and relied on quantitative methods only [58].	**I**

V = very good; A = adequate; D = doubtful; I = inadequate; N/A = No study available; “ see last rating above; bold = total ratings per section

^1^When the PROM was not developed in a sample representing the target population, the concept elicitation was not further rated

^2^PROMIS-scales developed together in one study are listed in combined rows

The involvement of the target population in concept elicitation was rated ‘inadequate’ (five PROMs) or ‘doubtful’ (five PROMs) for most PROMs. In some cases, no children were involved in the development studies (PAC-QOL, SQOLPOP, TAC-QOL). For other PROMs, methods were described insufficiently. For example, for the PedsQL modules, it remains unclear how they were derived from the previous PCQL.

For four instruments, no cognitive interviews were conducted (KINDL-R Oncology, PedsQL Generic, PedsQL Cancer, TACQOL), in another three cases, it remained ‘doubtful’ whether they were conducted in the target population (PedsQL Brain Tumor, QOLCC-7-12, SQOLPOP). The remaining studies solely investigated comprehensibility, whereas comprehensiveness was often not investigated (DISABKIDS, KIDSCREEN, KINDL-R Generic, PAC-QOL). All but one had to be rated as ‘doubtful’ or even ‘inadequate’ for comprehensiveness, mostly because it remained unclear whether the identified difficulties were addressed and because items were not appropriately (re-)tested in their final form. The PROMIS Pediatric Profile was the only instrument, for which ‘very good’ methods were applied and reporting was good. Nevertheless, it received an ‘adequate’ rating only, because most items were tested in five or six patients, while a ‘very good’ rating would have required seven or more patients per item.

The total rating for the development was based on the quality of concept elicitation and the quality of cognitive interview studies. The overall development was of ‘inadequate’ quality for eight PROMs and of ‘doubtful’ quality for another three PROMs. Only the PROMIS Pediatric Profile was informed by an ‘adequate’—almost ‘very good’—development procedure.

### Quality ratings of content validity studies

Quality ratings for content validity studies are provided in Table 3, including justifications for ratings other than ‘very good’ (V). Content validity studies were only conducted for three PROMs, the DISABKIDS, the KINDL-R Generic Module, and the QOLCC-7-12. For all three, quality was rated ‘inadequate’. The QOLCC-7-12 was only evaluated with five healthcare-experts, but no patients or caregivers were involved [65, 100]. For the DISABKIDS, only a few written comments by children and parents were taken into account, while focus groups were held with nurses [55]. Furthermore, it is questionable whether the comments resulted in any adaptations. In the study investigating the KINDL-R Generic Module, children were asked to rate the relevance and comprehensibility of the whole questionnaire, but not for each item individually [76].

**Table 3:** Quality ratings of content validity studies following the COSMIN methodology

PROM	Content validity studies								Justification for given ratings other than ‘very good’ (V)
	Patients			Experts		Parents^1^		Total content validation	
	Relevance	Comprehensiveness	Comprehensibility	Relevance	Comprehensiveness	Relevance	Comprehensiveness		
DISABKIDS	I	I	I	D	D	I	I	**I**	Validity assessment of the DCGM-37 in cancer patients primarily based on quantitative methods; focus groups on ‘feasibility’ with nurses, only a few written comments by parents or patients; suggestions for reformulations and to add filter-questions did not result in according adaptations [55].
KIDSCREEN	N/A	N/A	N/A	N/A	N/A	N/A	N/A	**I**	The assessment of psychometric properties of the final 52-item-version were exclusively based on quantitative methods [47, 48]. For the development of the shorter versions with 27 and 10 items, no information was found.
KINDL-R Generic	I	I	I	I	I	N/A	N/A	**I**	A CV study in children with chronic diseases (asthma, diabetes, not cancer), was ‘doubtful’, as they rated the comprehensibility and relevance of the questionnaire as a whole instead of single items [76]. Further validation based on quantitative methods only [49, 50].
KINDL-R Oncology	N/A	N/A	N/A	N/A	N/A	N/A	N/A	**I**	As it relied solely on quantitative methods to analyze reliability and construct validity, the paper by Ergin et al. [60] can not be taken into account as a content validity study.
PAC-QoL Child	N/A	N/A	N/A	N/A	N/A	N/A	N/A	**I**	No CV study available, but within a short correspondence Dr. Cataudella (Research Gate, 08/17/2020) announced that another paper on face validity and preliminary psychometrics is in preparation.
PedsQL Generic	N/A	N/A	N/A	N/A	N/A	N/A	N/A	**I**	No CV study available. In a study validating its Arabic version, Arabiat et al. [83] ask healthy children—interestingly not the children with cancer, who participated as well—to comment on the comprehensibility. However, no further information on methods or results reported.
PedsQL Cancer	N/A	N/A	N/A	N/A	N/A	N/A	N/A	**I**	No CV study available. Study on Chinese translation did not provide any relevant information [84]..
PedsQL Brain Tumor	N/A	N/A	N/A	N/A	N/A	N/A	N/A	**I**	Available studies on validating the PedsQL Brain Tumor Module relied on quantitative methods only [63, 81] and thus cannot be considered as CV studies.
PROMIS Ped Profile	N/A	N/A	N/A	N/A	N/A	N/A	N/A	**I**	No CV study in pediatric cancer patients available. Studies investigating PROMIS-scales in children with cancer relied on quantitative methods only [56, 57, 94–97, 104]. Also, studies on translations and cross-cultural comparison did not rely on qualitative methods [95, 98, 99]. Jones et al. [105] asked pediatric cancer survivors as well as their parents and clinicians for relevance ratings of items from four PROMIS Pediatric scales (i.e., fatigue, pain interference, psychological stress, positive affect). However, they did not report overall scores for relevance ratings, but only differences between child- and parent-ratings.
*Anxiety and depression*^*2*^	N/A	N/A	N/A	N/A	N/A	N/A	N/A	**I**	
*Fatigue*	N/A	N/A	N/A	N/A	N/A	N/A	N/A	**I**	
*Mobility and upper extremity*^*2*^	N/A	N/A	N/A	N/A	N/A	N/A	N/A	**I**	
*Pain Intensity*	N/A	N/A	N/A	N/A	N/A	N/A	N/A	**I**	
*Pain Interference*	N/A	N/A	N/A	N/A	N/A	N/A	N/A	**I**	
*Peer Relationships*	N/A	N/A	N/A	N/A	N/A	N/A	N/A	**I**	
QOLCC-7-12	I	I	I	D	D	N/A	N/A	**I**	CV was assessed within a Master Thesis, which is only available in Chinese [Huang, 2000 cited in 100]. Following scarce information provided in later studies [65, 100], no patients or parents were included. Five experts were involved to rate the relevance, analyzed with the content validity index (CVI). As it is unclear whether two researchers conducted the analysis, the quality remains ‘doubtful’.
SQOLPOP	N/A	N/A	N/A	N/A	N/A	N/A	N/A	**I**	Further validity and reliability testing by Kudubes and Bektas [67] relies on quantitative methods only and therefore cannot be considered as CV study. No other CV study available.
TACQOL	N/A	N/A	N/A	N/A	N/A	N/A	N/A	**I**	No CV study available. The reference study relied on quantitative methods only [102].

V, very good; A, adequate; D, doubtful; I, inadequate; N/A, no study available; “ see last rating above; bold = total ratings per section

^1^ This section was added to take the central role of parents in pediatric healthcare into account. As it is not required by the COSMIN Guidelines, ‘inadequate’ ratings were only given when parents were involved using ‘inadequate’ methods. If content validity studies were performed without parents, parent involvement was not rated

^2^ PROMIS-scales developed together in one study are listed in combined rows

### Rating of results and evidence grading

Following the COSMIN methodology, the development and content validity studies of mostly ‘doubtful’ or ‘inadequate’ quality can only provide ‘very low’ or ‘low’ evidence for the relevance, comprehensiveness, and comprehensibility of nearly all investigated PROMs. Only the PROMIS Pediatric Profile, with its ‘adequate’—almost ‘very good’—development procedure can rely on a ‘moderate’ evidence base for the three components of content validity. The quality of evidence for each PROM is displayed in Table 4, together with ratings of the results.

**Table 4:** Evidence grading and overall ratings for the relevance, comprehensiveness, and comprehensibility of the included patient-reported outcome measures (PROMs) for health-related quality of life (HRQOL) assessment in children with cancer

PROM	Relevance			Comprehensiveness			Comprehensibility		
	Rating of Results	Justification for rating of results	Quality of evidence	Rating of results	Justification for rating of results	Quality of evidence	Rating of results	Justification for rating of results	Quality of evidence
DISABKIDS 12/37	+	No cancer patients involved, but all items reflect concepts of the conceptual model for QOL provided by Anthony et al. [6].	Low	−	Mapping indicates underrepresentation of physical and cognitive issues (DCGM-12: 0 physical, 0 cognitive items DCGM-37: 3 physical, 1 cognitive items).	Low	−	Items, response options, and recall period are age-appropriate; but unclear if/how difficulties identified in studies were addressed [55, 69].	Low
KIDSCREEN 10/27/52	+	More than 90% of the items (except items on financial situation: KIDSCREEN 27: 2 items, KIDSCREEN 52: 3 items) match concepts of the QOL model by Anthony et al. [6].	Very low	−	Mapping indicates underrepresentation of physical and cognitive issues. KIDSCREEN 10, 27, 52 cover 0, 2, 2 physical and 1, 1, 1 cognitive items, respectively. Core symptoms like pain or nausea are missing	Very low	+	Studies state that no difficulties [48, 73]. This impression is shared by the reviewers.	Very low
Kid KINDL-R Generic	+	When asked about the relevance of the whole questionnaire, 80% report perceiving it as very relevant or relevant [76]. All items could be mapped onto the model of QOL by Anthony et al. [6].	Low	−	Mapping indicates underrepresentation of physical (3 items) and cognitive (2 items) issues in the generic tool, which might explain why 20% of chronically ill children did not perceive the questionnaire as a whole as relevant [76].	Low	+	“95.5% of the chronically ill children found the questions easily understandable” [76].	Low
Kid-Kiddo KINDL-R Oncology	+	All items could be mapped onto the QOL model by Anthony et al. [6].	Very low	−	Mapping indicates that the tool is not comprehensive. Cognitive issues are not assessed at all. From the psychological domain, only treatment burden (“bothered”; 12 items) and moodiness (2 items) are assessed. Neither other forms emotional distress nor positive mental health aspects are covered.	Very low	−	The reviewers rated the comprehensibility as insufficient due to very complex response-options. Many items require more than one response: For symptoms, children must indicate frequency and the resulting burden. For treatment- or procedure-related issues, a conditional item is followed by a frequency and a burden rating.	Very low
PAC-QoL Child	+	Items mapped well onto the conceptual model by Anthony et al. [6], except four items covering involvement in care, which was added as a new subdomain in this review.	Very low	+	All domains and nearly all subdomains (except self-esteem and treatment-burden) are covered.	Very low	−	Results of studies available at the time indicate insufficient comprehensibility [62].	Very low
PedsQL Generic Core Scale 4.0	+	All items cover issues reflecting the QOL model by Anthony et al. [6].	Very low	−	No items assess positive psychological functioning, self-esteem, or body image.	Very low	+	Reviewers' ratings indicate sufficient comprehensibility because instructions, items, and response-options are considered age-appropriate.	Very low
PedsQL Cancer Module 3.0	+	All items cover issues reflecting the QOL model by Anthony et al. [6].	Very low	−	No items on positive psychological functioning are assessed. The social domain does only contain items on involvement in care, whereas the social context of family, peers, school, and leisure time is missing.	Very low	+	Reviewers' ratings indicate sufficient comprehensibility because instructions, items, and response-options are considered age-appropriate.	Very low
PedsQL Brain Tumor Module	+	All items cover issues reflecting the QOL model by Anthony et al. [6].	Very low^1^	−	No items on positive psychological functioning or social domain.	Very low^1^	+	Reviewers' ratings indicate sufficient comprehensibility because instructions, items, and response-options are considered age-appropriate.	Very low^1^
PROMIS Ped Profile 25/37/49	+	All items cover issues reflecting the QOL model by Anthony et al. [6]. Jones et al. [105] did not report any general relevance issues when comparing child- and parent ratings on relevance.	Moderate	±	Healthy children and pediatric asthma patients had nothing to add [85]. However, this might not be representative for children with cancer. (Children with chronic kidney or Crohn’s disease and adult cancer patients had disease-specific issues to add; see [106, 107]). Mapping indicates that cognitive as well as positive psychological issues are missing. Even though the physical domain takes approximately the half of items, none covers nausea, a core symptom in oncology.	Moderate	+	PROMIS is the only inventory reporting comprehensibility for items, response-options, and instructions separately and results indicate very good comprehensibility [85].	Moderate
QOLCC-7-12	+	Yeh et al. [64] report that besides quantitative criteria, “items were retained if at least two pediatric oncology specialists or three patients from the qualitative study considered them clinically important” (p.163). Most items mapped well onto the model by Anthony et al. [6], except three items on knowledge about cancer and three items on involvement in care—aspects which were added as new subdomains.	Very low	+	Mapping indicates that all relevant domains are represented, even though the physical domain takes a smaller proportion than in other cancer-specific tools.	Very low	+	Important study (Huang and Yeh, 2000 cited in [100]) not accessible due to language barriers (request for English abstract unanswered). Reviewers' ratings indicate sufficient comprehensibility, because item wording was considered age-appropriate. No information about recall-period or response-options available.	Very low
SQOLPOP	?	In case of ‘very low’ evidence, the rating of results should rely on reviewers’ ratings only. However, as no review copy or item list was available, it was impossible to make any ratings.	Very low	?	In case of ‘very low’ evidence, the rating of results should rely on reviewers’ ratings only. However, as no review copy or item list was available, it was impossible to make any ratings.	Very low	?	In case of ‘very low’ evidence, the rating of results should rely on reviewers’ ratings only. However, as no review copy or item list was available, it was impossible to make any ratings.	Very low
TACQOL	+	All items cover issues reflecting the QOL model by Anthony et al. [6].	Very low	+	All domains of the QOL model by Anthony et al. [6] are covered and take a reasonable proportion.	Very low	+	Following the impression of Vogels et al. [58] that the questionnaire is “self-explanatory” (p. 460), comprehensibility was rated sufficient.	Very low

+, sufficient; −, insufficient; ±, inconsistent; ?, indeterminate (only if no evidence from studies and no access to review copy for own ratings)

VL = very low (PROM development study of ‘inadequate’ quality AND only content validity studies of ‘inadequate’ quality or no content validity studies (only reviewers’ ratings))

L = low (PROM development study of ‘doubtful’ quality AND only content validity studies of ‘inadequate’ quality or no content validity studies)

M = moderate (PROM development study of ‘very good’ or ‘adequate’ quality AND only content validity studies of ‘inadequate’ quality or no content validity studies; OR at least one content validity study of ‘doubtful’ quality)

^1^Following the COSMIN guidelines, evidence was ‘low’, because the development procedure is ‘doubtful’ and no content validity study is available. However, no study results are available, so that rating of results is solely based on reviewers’ ratings. Thus, the evidence was down-graded to ‘very low’. This is in line with a current review by Bull et al. [37], who state that no information on the content validity is provided for the PedsQL Brain Tumor Module.

Due to the ‘very low’ evidence for most PROMs, the ratings often rely on reviewers’ ratings. As no review copy was available for the SQOLPOP, only ‘indeterminate’ ratings could be given for this instrument. For all other measures, ratings of results for relevance and comprehensiveness were based strictly on the content categorization described before. Relevance was rated as ‘sufficient’ because all items could be mapped onto the conceptual model of HRQOL. However, the comprehensiveness of seven PROMs was rated as ‘insufficient’, mostly because cognitive issues or positive psychological functioning were missing.

As all instruments have age-appropriate recall-periods and response-options, reviewers’ comprehensibility ratings were positive and/or followed the study results. Only for the KINDL-R Oncology Module, did reviewers rate the comprehensibility as ‘insufficient’, because its design is considerably complex. In this PROM, some items require three responses: For symptoms, children must indicate frequency and the resulting burden. For treatment- or procedure-related issues, a conditional item is followed by frequency and burden ratings.

## Discussion

The quality assessment of development, cognitive interview, and content validity studies showed that none of the investigated PROMs has a solid evidence base for its content validity. For most instruments, evidence is ‘very low’, only the PROMIS Pediatric Profile is based on ‘moderate’ evidence. Overall, the scarce evidence available indicates that the PROMs cover relevant issues, while evidence for comprehensiveness and comprehensibility is partly inconsistent or indicates that these have not been sufficiently fulfilled.

### Methodological shortcomings and possible explanations

The reasons for this low evidence level can be found in the study design, methodological quality, and insufficient reporting. As already stated by Klassen et al. [31], patients were not sufficiently involved. Guidelines on patient involvement in PROM development as well as reporting guidelines did only appear after most instruments had been developed. Thus, the developers of the investigated PROMs could not yet benefit from their guidance. The concept of content validity in particular has not been clearly defined for a long time.

#### Missing qualitative studies and patient involvement

Most of the PROMs were developed in the 1990s or early 2000s, before the publication of milestone policies by the European Medicines Agency (EMA) [108] and the American Food and Drug Administration (FDA) [109] and methodological guidelines on PROM development or content validity around 2010, e.g., by the International Society for Pharmacoeconomics and Outcomes Research Patient Reported Outcome Good Research Practices Task Force (ISPOR PRO) [24–26, 110] or the PROMIS developers [85, 86]. This might explain poor or inconsistent methods and reporting. However, missing or ‘inadequate’ development studies could be compensated by qualitative content validity studies to strengthen the evidence for existing tools. As an example, the content validity of the most widely used adult cancer questionnaire, the EORTC QLQ-C30, is currently being evaluated with adult [111] and adolescent cancer patients [112]. For the pediatric PROMs included in the present review, almost no content validity studies were available.

Lacking qualitative evidence, investigators take the mere use of questionnaires as an indicator of content validity. For example, Arabiat et al. state that “Face and content validity were assumed because the PedsQL™ (4.0) is widely used and reported in quality of life research” [83]. Despite strong recommendations for patient involvement, there are several barriers for qualitative research. Applying qualitative methods is partly a question of resources (i.e., financial means, infrastructure, collaborations, expertise, etc.). For example, Petersen et al., who interviewed children during the development procedure of the DISABKIDS, concluded that “these techniques are a helpful method. Nevertheless, the amount of time necessary to carry this out and analyze it is a weakness of this approach” [69]. Despite these challenges, qualitative methods are crucial, because content validity is a question of heuristics that cannot be resolved by quantitative methods.

#### Missing clarity about the concept of content validity

Another reason for missing research on content validity might be that this measurement property has been the subject of scientific dispute [113]. Following critique from modern test theory, guidelines seemingly struggled to redefine the concept and to identify methods for its assessment [113, 114]. It is only in the latest version of the COSMIN methodology that content validity is clearly described by the three components of relevance, comprehensiveness, and comprehensibility, and that corresponding standards and criteria are defined [21, 22]. This new and clear definition and the high requirements of the recent COSMIN guidelines make a considerable difference. Wayant et al. [35], who used the new methodology, found the same lack of evidence highlighted by our review. This is in contrast with reviews based on the older version, which came to very positive results [e.g., 34].

As the operationalization of content validity by relevance, comprehensibility, and comprehensiveness is still young, studies so far have seldom covered all three components separately and equally. For example, Kudubes and Bektas [67] asked health-care professionals only to rate how much change was needed for each item, without specifying what kind of change was required and why. If studies made a distinction between the three components, comprehensiveness was less often investigated compared to relevance and comprehensibility. This is in line with a recent review of studies on measurement properties of PROMs, which found that 77.8% of the studies assessed relevance, 48.2% evaluated comprehensibility, and only 3.7% focused on comprehensiveness [115].

When it comes to comprehensibility, there is again a lack of differentiation. Wayant et al. [35] state that instructions were not investigated for any of the PROMs included in their review; rather, the studies focused solely on items. In our review, the PROMIS Pediatric Profile is the only tool for which items, instructions, response-options, and recall-periods were assessed separately [85]. For the KINDL Generic Module, which was developed a decade earlier, comprehensibility was not even rated per item, but for the whole questionnaire [76].

#### ‘Doubtful’ ratings of study quality due to poor reporting

Not only is there a lack of qualitative studies of high quality for assessing content validity, but most ‘doubtful’ ratings were given due to insufficient reporting. In several cases, development and cognitive interview studies were only briefly described in a paragraph of a later study focusing on quantitative validity or reliability testing. Such shortcomings in reporting of qualitative methods in PROM development are a well-known problem and not specific to the field of pediatric oncology [116].

The recently published COSMIN reporting guideline will hopefully improve the situation [117]. However, it gives only very loose rules for content validity studies, defining *what* must be reported. It does not provide guidance on *how much detail* is required to meet the criteria of the COSMIN methodology for assessing content validity. Therefore, it might be useful to also have this methodology in mind when developing a new instrument. Even though Gagnier et al. differentiate clearly between the scopes of the two guidelines [117], it would surely help to prepare, conduct, and report future research more effectively and to provide more solid evidence.

### Limitations and challenges of applying the COSMIN methodology on content validity assessment

We are aware that the search strategy underlying this review was limited. The search was conducted in only one database, PubMed, and did not rely on the extensive search filter by COSMIN [118]. This filter, however, is designed to find studies reporting all psychometric properties and not specifically content validity. Thus, the results would have exceeded the scope of our review. That no further PROMs could be identified through cross-checking with very comprehensive reviews [44, 45] indicates that our search was sufficiently fit for identifying relevant PROMs. Corresponding development and content validity studies are usually referred to as primary citations. Beyond that, we conducted additional searches and contacted PROM designers and authors to make sure that no relevant studies were missed.

While the COSMIN methodology is the current gold standard for assessing the quality criteria of PROMs, its application was partly challenging. Not only is the reporting inconsistent and insufficient, but the differentiation between cognitive interview and content validity studies is sometimes difficult to make. Furthermore, the COSMIN guidelines propose rating each subscale separately [22]. This was rarely possible, because most of the multidimensional PROMs were developed as a whole and the information was not given per subscale. Even for the PROMIS Pediatric Profile, for which subscales were developed separately, not all steps and results were reported for each subscale in detail. These uncertainties led to many ‘doubtful’ ratings. Since the COSMIN methodology follows the worst-score-counts-principle, one ‘doubtful’ rating results in a ‘doubtful’ overall rating. This principle could be criticized for being too strict, as less relevant deficiencies could outweigh more important standards that were well met.

The situation is further complicated because the guidelines were not developed for pediatric tools and do not provide any advice on how to consider evidence provided by caregivers. We tried to resolve this by adding the standards required for expert involvement in content validity studies to take caregiver interviews into account. One could argue that caregivers’ input should also have been considered in concept elicitation or cognitive interview studies. However, as caregiver- and patient-report often differ considerably, we decided to not systematically consider input from caregivers during these steps—in exactly the same way that the opinions of health-care professionals are ignored at this point following the COSMIN guidelines.

### Conclusion and implications

Following the COSMIN methodology, this systematic review showed that there is only fragile evidence for the content validity of PROMs for HRQOL in children with cancer. Only the PROMIS Pediatric Profile has a ‘moderate’ level of evidence. Results indicate that it covers relevant issues and is comprehensible. Its comprehensiveness could be improved by adding further pediatric PROMIS scales (e.g., cognitive function, meaning and purpose, life satisfaction, positive affect) [43]. Thus, among the investigated PROMs, the Pediatric PROMIS Profile is recommended. However, this instrument is not disease-specific, and it might be worthwhile conducting a qualitative content validity study in children with cancer.

This lack of evidence can be explained by several factors: Most investigated instruments were developed before the publication of milestone policies and guidelines. Learning from the strengths and limitations of said previous PROM developments, these guidelines set new methodological standards. Content validity, in particular, was only clearly defined in the latest version of the COSMIN methodology. While it is, therefore, understandable that previous projects did not fulfill all required standards, PRO and HRQOL research in pediatric oncology should still try to catch up with the scientific and methodological progress of the last decade.

Therefore, we argue that further efforts are needed to provide PROMs for HRQOL assessment in children with cancer that are based on solid evidence. This could include the development of new instruments, as well as performing content validity studies to strengthen the evidence for already-existing PROMs. In each case, it is strongly recommended that existing guidelines on qualitative methods and reporting standards for these study types be adhered to. Within the EORTC QLG, we are currently developing an HRQOL questionnaire for children with cancer [119]. Following the EORTC QLG module development guidelines [23], this involves not only a literature review [45], but also in-depth interviews with children with cancer, their parents, and health-care professionals.

## Supplementary Information

**Additional file 1.** PRISMA Checklist.

**Additional file 2.** Categorization Rules.

**Additional file 3.** Adaptations to Complement the Model of Health-Related Quality of Life.

**Additional file 4.** List of items per Patient-Reported Outcome Measure (PROM) covering the various domains, subdomains and identifying concepts of health-related quality of life.

## Data Availability

The authors declare that the relevant data supporting our findings is provided in the article and the supplementary files. For further requests, please contact the corresponding author.
