# Supplementary material for: A critical evaluation of the content validity of patient-reported outcome measures assessing health-related quality of life in children with cancer: a systematic review
Source: J Patient Rep Outcomes. 2023 Jan 19;7:2. doi: 10.1186/s41687-023-00540-8 (PMC9851583; doi:10.1186/s41687-023-00540-8)
Supplement: Supplementary file 2 — Additional file 2. Categorization Rules. [file 41687_2023_540_MOESM2_ESM.docx]

**Supplement 2: Categorization Rules**

In order to clarify the categorizations and to proceed consistently, several rules have been defined a priori. Most importantly, interference items were categorized depending on the cause (grammatical explanans). Only if the cause was the health condition in general, the item was categorized depending on the effect (grammatical explanandum).

Activities were rated as physical activity if they are described as basic physical functioning without any reference to pleasure or any social context (i.e., domain: physical health; subdomain: physical function; identifying concept: physical activity or mobility). Activities that are considered to be done for pleasure were rated as referring to recreation / leisure or sports and therefore considered as part of social function (i.e., domain: social health; subdomain: social function: identifying concept: recreation/leisure or sports). If items described the activities in a specific social context (e.g., “with other kids”), the items were rated as social function in the peer group (i.e., domain: social health; subdomain: social function; identifying concept: peers).

We avoided to use the subdomain of behavior, because the behaviors described in items could always be considered as social behavior and thus was categorized as describing social relationships or social function.
