# Supplementary material for: A critical evaluation of the content validity of patient-reported outcome measures assessing health-related quality of life in children with cancer: a systematic review
Source: J Patient Rep Outcomes. 2023 Jan 19;7:2. doi: 10.1186/s41687-023-00540-8 (PMC9851583; doi:10.1186/s41687-023-00540-8)
Supplement: Supplementary file 3 — Additional file 3. Adaptations to Complement the Model of Health-Related Quality of Life. [file 41687_2023_540_MOESM3_ESM.docx]

**Supplement 3: Adaptations to Complement the Model of Health-Related Quality of Life**

The general health domain was complemented by subscales for *children’s knowledge about their illness* and the *interference with daily life*. The latter might also be placed in the social functioning subdomain, but as the items were quite broad in focus, we decided to add them into the general health domain.

The psychological health domain was complemented by a subdomain for items referring to explicitly *treatment-related burdens* (e. g. procedure-related anxiety; being bothered by different treatments). This subdomain can be considered as a specification of the emotional distress subdomain.

A third subdomain was added in the social health domain and brings in an important issue that was not represented in the model by Anthony et al. (2014) so far: *Involvement in Care and Treatment*. This subdomain contains items on the children’s possibility to express their own perspective, needs and questions to HCPs, but also if the children are informed about their health status and treatment, and if they can participate and are involved in shared decision-making processes.

In Figure 1, the *cognitive subdomain is presented separately* from the other psychological subdomains, which are referred to as “emotional”. This decision is based on the reasoning that cognitive issues take a special role within the psychological domain: They do not directly reflect emotional well-being and they could also contain or at least be influenced by physical, i.e., neurological issues. This division is supported by other QOL models (e.g. Hinds, 1990).

Five items referring to the *financial situation* of the children were summarized in a new domain for “other aspects”. It could also be placed in the social or general health domain. However, we considered financial issues less relevant for HRQOL assessment in children with cancer and therefore decided to subsume it as other.
