# Supplementary material for: A critical evaluation of the content validity of patient-reported outcome measures assessing health-related quality of life in children with cancer: a systematic review
Source: J Patient Rep Outcomes. 2023 Jan 19;7:2. doi: 10.1186/s41687-023-00540-8 (PMC9851583; doi:10.1186/s41687-023-00540-8)
Supplement: Supplementary file 4 — Additional file 4. List of items per Patient-Reported Outcome Measure (PROM) covering the various domains, subdomains and identifying concepts of health-related quality of life. [file 41687_2023_540_MOESM4_ESM.docx]

**Supplement 4: List of items per Patient-Reported Outcome Measure (PROM) covering the various domains, subdomains and identifying concepts of health-related quality of life.**

|  | **DISABKIDS DCGM 12** | **DISABKIDS DCGM 37** | **KIDSCREEN 10** | **KIDSCREEN 27** | **KIDSCREEN 52** | **KINDL Kid Core** | **KINDL Oncology** | **PAC-QOL (provisional)** | **PedsQL Brain Tumor** | **PedsQL Cancer** | **PedsQL Generic** | **PROMIS Ped Profile 25** | **PROMIS Ped Profile 37** | **PROMIS Ped Profile 49** | **QOLCC** | **TACQOL-CF** |
| --- | --- | --- | --- | --- | --- | --- | --- | --- | --- | --- | --- | --- | --- | --- | --- | --- |
| **General Health** | **2** | **4** | **3** | **3** | **3** | **1** | **1** | **1** | **1** | **1** |  |  |  |  | **3** |  |
| **Health Perception** |  |  | **3** | **3** | **3** | **1** | **1** | **1** | **1** | **1** |  |  |  |  |  |  |
| future |  |  |  |  |  |  | 1 | 1 | **1** | **1** |  |  |  |  |  |  |
| present |  |  | 3 | **3** | **3** | 1 |  |  |  |  |  |  |  |  |  |  |
| **Interference with Daily**  **Life*** | **2** | **4** |  |  |  |  |  |  |  |  |  |  |  |  |  |  |
| **Knowledge about  Illness*** |  |  |  |  |  |  |  |  |  |  |  |  |  |  | **3** |  |
| **Physical Health** |  | **3** |  | **2** | **2** | **3** | **8** | **15** | **11** | **7** | **8** | **12** | **17** | **23** | **6** | **19** |
| **Physical Function** |  | **2** |  | **2** | **2** | **1** | **4** | **7** | **4** | **1** | **6** | **3** | **5** | **7** | **4** | **11** |
| Appetite* |  |  |  |  |  |  | 2 | 1 |  |  |  |  |  |  |  |  |
| Dexterity |  |  |  |  |  |  |  | 1 | **1** |  |  |  | **1** | **1** |  | 2 |
| Extremity Function* |  |  |  |  |  |  |  | 1 | **2** |  |  |  |  | **1** |  |  |
| Mobility |  |  |  |  |  |  | 1 |  |  |  | 1 | **2** | **2** | **3** | 1 | 3 |
| Physical Activity |  | **1** |  | **2** | **2** |  | 1 |  |  |  | 2 | **1** | **1** | **1** | 1 | 2 |
| Self-Care |  |  |  |  |  |  |  | 1 |  |  | 1 |  |  |  | 1 | 4 |
| Senses |  |  |  |  |  |  |  | 1 | **1** | **1** |  |  |  |  |  |  |
| Sleep |  | **1** |  |  |  |  |  | 1 |  |  | 1 |  |  |  |  |  |
| Speech |  |  |  |  |  |  |  | 1 |  |  |  |  |  |  |  |  |
| Strength |  |  |  |  |  | 1 |  |  |  |  | 1 |  | **1** | **1** | 1 |  |
| **Symptom** |  | **1** |  |  |  | **2** | **4** | **8** | **7** | **6** | **2** | **9** | **12** | **16** | **2** | **8** |
| Diet |  |  |  |  |  |  | 1 |  |  |  |  |  |  |  |  |  |
| Dizzy |  |  |  |  |  |  |  |  |  |  |  |  |  |  |  | 1 |
| Fatigue |  | **1** |  |  |  | 1 |  | 1 |  |  | 1 | **4** | **5** | **7** |  | 2 |
| Nausea |  |  |  |  |  |  | 2 | 1 | **3** | **3** |  |  |  |  | 1 | 1 |
| Pain |  |  |  |  |  | 1 |  | 2 | **4** | **3** | 1 | **5** | **7** | **9** | 1 | 2 |
| Respiratory Changes |  |  |  |  |  |  |  | 1 |  |  |  |  |  |  |  |  |
| Other* |  |  |  |  |  |  | 1 | 3 |  |  |  |  |  |  |  | 2 |
| **Psychological Health** | **8** | **19** | **3** | **8** | **19** | **11** | **14** | **14** | **12** | **17** | **7** | **8** | **12** | **16** | **19** | **24** |
| **Cognitive** |  | **1** | **1** | **1** | **1** | **2** |  | **1** | **7** | **6** | **3** |  |  |  | **5** | **8** |
| Attention |  |  | 1 | **1** | **1** |  |  |  | **1** | **1** | 1 |  |  |  | 2 |  |
| Communication |  |  |  |  |  |  |  |  |  | 1 |  |  |  |  |  | 2 |
| Concentration |  | **1** |  |  |  |  |  |  |  |  |  |  |  |  |  | 1 |
| Learning |  |  |  |  |  |  |  |  | **1** | **0** |  |  |  |  |  | 1 |
| Problem-Solving |  |  |  |  |  | 1 |  |  | **2** | **2** |  |  |  |  |  | 1 |
| Remembering |  |  |  |  |  |  |  | 1 | **1** | **1** | 1 |  |  |  | 2 |  |
| General* |  |  |  |  |  | 1 |  |  | **2** | **1** | 1 |  |  |  | 1 | 3 |
| *… see next page for continuation of this table.* | | | | | | | | | | | | | | | | |

*Continued Supplement 4:*

|  | **DISABKIDS DCGM 12** | **DISABKIDS DCGM 37** | | **KIDSCREEN 10** | | **KIDSCREEN 27** | | **KIDSCREEN 52** | | **KINDL Kid Core** | | **KINDL Kid Oncology** | | **PAC-QOL (provisional)** | | **PedsQL Brain Tumor** | | **PedsQL Cancer** | | **PedsQL Generic** | | **PROMIS Ped Profile 25** | | **PROMIS Ped Profile 37** | | **PROMIS Ped Profile 49** | **QOLCC** | **TACQOL-CF** |
| --- | --- | --- | --- | --- | --- | --- | --- | --- | --- | --- | --- | --- | --- | --- | --- | --- | --- | --- | --- | --- | --- | --- | --- | --- | --- | --- | --- | --- |
| **Emotional Distress** | **3** | **6** | | **2** | | **3** | | **6** | | **4** | | **2** | | **8** | |  | |  | | **4** | | **8** | | **11** | | **15** | **5** | **8** |
| Afraid |  |  | |  | |  | |  | |  | |  | | 2 | |  | |  | | 1 | |  | |  | |  |  |  |
| Angry |  | **1** | |  | |  | |  | |  | |  | | 1 | |  | |  | | 1 | |  | |  | |  | 1 | 2 |
| Annoyed |  |  | |  | |  | | **1** | |  | |  | |  | |  | |  | |  | |  | |  | |  |  |  |
| Anxious |  |  | |  | |  | |  | | 1 | |  | |  | |  | |  | |  | | **1** | | **2** | | **3** |  | 1 |
| Bored |  |  | |  | |  | |  | | 1 | |  | |  | |  | |  | |  | |  | |  | |  |  |  |
| Discouraged |  |  | |  | | **1** | | **1** | |  | |  | |  | |  | |  | |  | |  | |  | |  | 1 |  |
| Frustrated |  |  | |  | |  | | **1** | |  | |  | |  | |  | |  | |  | | **1** | | **1** | | **1** |  |  |
| Irritable |  |  | |  | |  | |  | |  | |  | |  | |  | |  | |  | |  | |  | |  |  | 1 |
| Jealous |  |  | |  | |  | |  | |  | |  | |  | |  | |  | |  | |  | |  | |  |  | 1 |
| Lonely* | 1 | **1** | | 1 | | **1** | | **1** | | 1 | |  | | 1 | |  | |  | |  | | **1** | | **1** | | **2** | 1 |  |
| Miserable |  |  | |  | |  | |  | |  | |  | |  | |  | |  | |  | | **1** | | **1** | | **1** |  |  |
| Moody |  |  | |  | |  | |  | |  | | 2 | | 1 | |  | |  | |  | |  | |  | |  |  |  |
| Nervous* |  |  | |  | |  | |  | |  | |  | | 1 | |  | |  | |  | | **1** | | **1** | | **1** |  |  |
| Sad | 2 | **2** | | 1 | | **1** | | **1** | |  | |  | | 1 | |  | |  | | 1 | | **1** | | **2** | | **3** | 1 | 2 |
| Stressed |  |  | |  | |  | | **1** | |  | |  | |  | |  | |  | |  | |  | |  | |  |  |  |
| Worried |  | **2** | |  | |  | |  | | 1 | |  | | 1 | |  | |  | | 1 | | **2** | | **3** | | **4** | 1 | 1 |
| **Treatment Burden*** | **2** | **4** | |  | |  | |  | |  | | **12** | |  | | **5** | | **8** | |  | |  | |  | |  | **4** |  |
| Anxiety* |  |  | |  | |  | |  | |  | |  | |  | | **3** | | **6** | |  | |  | |  | |  | 2 |  |
| Bothered* | 2 | **3** | |  | |  | |  | |  | | 12 | |  | |  | |  | |  | |  | |  | |  |  |  |
| Worried* |  | **1** | |  | |  | |  | |  | |  | |  | | **2** | | **2** | |  | |  | |  | |  | 2 |  |
| **Positive Psychological  Function** |  | **2** | |  | | **3** | | **6** | | **1** | |  | | **4** | |  | |  | |  | |  | |  | |  |  | **8** |
| Benefit Finding |  |  | |  | |  | | **1** | |  | |  | | 3 | |  | |  | |  | |  | |  | |  |  |  |
| Calm |  |  | |  | |  | |  | |  | |  | |  | |  | |  | |  | |  | |  | |  |  | 1 |
| Confident |  | **1** | |  | |  | |  | |  | |  | | 1 | |  | |  | |  | |  | |  | |  |  | 1 |
| Happy |  | **1** | |  | | **2** | | **3** | | 1 | |  | |  | |  | |  | |  | |  | |  | |  |  | 5 |
| Satisfied |  |  | |  | | **1** | | **2** | |  | |  | |  | |  | |  | |  | |  | |  | |  |  | 1 |
| **Self Esteem** | **3** | **5** | |  | | **1** | | **2** | | **4** | |  | |  | |  | |  | |  | |  | | **1** | | **1** | **1** |  |
| Feel different | 2 | **2** | |  | |  | |  | | 1 | |  | |  | |  | |  | |  | |  | |  | |  |  |  |
| Feel good about  abilities^+^ | 1 | **2** | |  | |  | | **1** | |  | |  | |  | |  | |  | |  | |  | | **1** | | **1** |  |  |
| Feel good about self |  | **1** | |  | | **1** | | **1** | | 1 | |  | |  | |  | |  | |  | |  | |  | |  | 1 |  |
| Feel proud |  |  | |  | |  | |  | | 2 | |  | |  | |  | |  | |  | |  | |  | |  |  |  |
| **Body Image** |  | **1** | |  | |  | | **4** | |  | |  | | **1** | |  | | **3** | |  | |  | |  | |  | **4** |  |
| Personal Appearance |  | **1** | |  | |  | | **4** | |  | |  | | 1 | |  | | **3** | |  | |  | |  | |  | 4 |  |
|  |  | |  | |  | |  | |  | |  | |  | |  | |  | |  | |  | |  | |  |  |  |  |
| *… see next page for continuation of this table.* | | | | | | | | | | | | | | | | | | | | | | | | | | | | |

*Continued Supplement 4:*

|  | **DISABKIDS DCGM 12** | **DISABKIDS DCGM 37** | **KIDSCREEN 10** | **KIDSCREEN 27** | **KIDSCREEN 52** | **KINDL Kid Core** | **KINDL Oncology** | **PAC-QOL (provisional)** | **PedsQL Brain Tumor** | **PedsQL Cancer** | **PedsQL Generic** | **PROMIS Ped Profile 25** | **PROMIS Ped Profile 37** | **PROMIS Ped Profile 49** | **QOLCC** | **TACQOL-CF** |
| --- | --- | --- | --- | --- | --- | --- | --- | --- | --- | --- | --- | --- | --- | --- | --- | --- |
| **Social Health** | **2** | **11** | **5** | **12** | **25** | **9** | **5** | **12** |  | 2 | **8** | **5** | **8** | **10** | **6** | **13** |
| **Relationship** | **1** | **6** | **2** | **8** | **17** | **7** | **4** | **3** |  |  | **2** | **4** | **5** | **7** | **2** | **6** |
| Family |  |  | 1 | **3** | **6** | 4 |  | 1 |  |  |  |  |  |  | 2 | 4 |
| Peers | 1 | **3** | 1 | **4** | **9** | 3 | 3 |  |  |  | 2 | **4** | **5** | **7** |  | 2 |
| Teachers |  | **1** |  | **1** | **2** |  |  |  |  |  |  |  |  |  |  |  |
| Others in General* |  | **2** |  |  |  |  | 1 | 2 |  |  |  |  |  |  |  |  |
| **Social Function** | **1** | **5** | **3** | **4** | **8** | **2** | **1** | **5** |  |  | **6** | **1** | **3** | **3** | **1** | **7** |
| Family |  |  |  |  |  |  |  | 1 |  |  |  |  |  |  |  |  |
| Peers |  | **1** |  |  | **1** |  |  | 1 |  |  | 3 |  | **2** | **2** |  | 2 |
| Recreation and   Leisure | 1 | **3** | 2 | **2** | **4** |  |  | 1 |  |  |  |  |  |  |  | 3 |
| School |  |  | 1 | **2** | **3** | 2 | 1 | 1 |  |  | 2 |  |  |  |  | 1 |
| Sports |  |  |  |  |  |  |  |  |  |  | 1 | **1** | **1** | **1** | 1 | 1 |
| Others in General* |  | **1** |  |  |  |  |  | 1 |  |  |  |  |  |  |  |  |
| **Involvement in  Care*** |  |  |  |  |  |  |  | **4** |  | **2** |  |  |  |  | **3** |  |
| able to articulate own  perspective/needs* |  |  |  |  |  |  |  | 1 |  | **2** |  |  |  |  | 2 |  |
| being informed* |  |  |  |  |  |  |  | 2 |  |  |  |  |  |  | 1 |  |
| shared decision-  making* |  |  |  |  |  |  |  | 1 |  |  |  |  |  |  |  |  |
| **Other Aspects*** |  |  |  | **2** | **3** |  |  |  |  |  |  |  |  |  |  |  |
| **Financial*** |  |  |  | **2** | **3** |  |  |  |  |  |  |  |  |  |  |  |
| **Sum of items / PROM** | **12** | **37** | **11** | **27** | **52** | **24** | **28** | **42** | **24** | **27** | **23** | **25** | **37** | **49** | **34** | **56** |
| *added domain / subdomain / identifying concept, complementing the model from Anthony et al. (2014).  ^+^ less differentiated compared to the model by Anthony et al. (2014) who specified various abilities / contexts. | | | | | | | | | | | | | | | | |
